# Pre-pregnancy migraine diagnosis, medication use, and spontaneous abortion: a prospective cohort study

**DOI:** 10.1186/s10194-022-01533-6

**Published:** 2022-12-20

**Authors:** Holly M. Crowe, Amelia K. Wesselink, Lauren A. Wise, Susan S. Jick, Kenneth J. Rothman, Ellen M. Mikkelsen, Henrik T. Sørensen, Elizabeth E. Hatch

**Affiliations:** 1 Harvard T.H. Chan School of Public Health, Department of Epidemiology, 677 Huntington Avenue, Boston, MA 02115 USA; 2 Boston University School of Public Health, Department of Epidemiology, 715 Albany Street, Boston, MA 02118 USA; 3 Aarhus University, Department of Clinical Epidemiology, Olof Palmes Allé 43-45, DK-8200 Aarhus N, Denmark

**Keywords:** Migraine, Triptans, Epidemiology, Pregnancy, Spontaneous abortion, Miscarriage

## Abstract

**Background:**

Migraine is common among females of reproductive age (estimated prevalence:17–24%) and may be associated with reproductive health through underlying central nervous system excitability, autoimmune conditions, and autonomic dysfunction. We evaluated the extent to which pre-pregnancy migraine diagnosis and medication use are associated with risk of spontaneous abortion (SAB).

**Methods:**

We analyzed data from a preconception study of pregnancy planners (2013–2021). Eligible participants self-identified as female, were aged 21–45 years, resided in the USA or Canada, and conceived during follow-up (*n* = 7890). Participants completed baseline and bimonthly follow-up questionnaires for up to 12 months or until a reported pregnancy, whichever occurred first. Pregnant participants then completed questionnaires during early (~ 8–9 weeks) and late (~ 32 weeks) gestation. We defined migraineurs as participants who reported a migraine diagnosis or use of a medication to treat migraine. Preconception questionnaires elicited migraine medication use during the past 4 weeks, and SAB on follow-up and pregnancy questionnaires. We used Cox regression models with gestational weeks as the time scale to estimate hazard ratios (HRs) and 95% confidence intervals (CIs) for associations among preconception migraine, migraine medication use, and SAB, controlling for potential demographic, medical, and lifestyle confounders.

**Results:**

Nineteen percent of study pregnancies ended in SAB. History of migraine before conception was not appreciably associated with SAB risk (HR = 1.03, 95% CI: 0.91–1.06). Use of any migraine medication was associated with a modest increase in SAB risk overall (HR = 1.14, 95% CI: 0.96–1.36). We observed the greatest increase in risk among those taking migraine medications daily (HR = 1.38, 95% CI: 0.81–2.35) and those taking prescription migraine prophylaxis (HR = 1.43, 95% CI: 0.72–2.84) or combination analgesic and caffeine medications (HR = 1.42, 95% CI: 0.99–2.04).

**Conclusions:**

Migraine medication use patterns suggesting greater underlying migraine severity were associated with increased risk of SAB. This research adds to the limited information available on the reproductive effects of migraine.

## Background

Migraine is common among individuals of reproductive age, with an estimated prevalence of 17% in females aged 18–29 years and 24% in females aged 30–39 years [1, 2]. Migraine can deleteriously affect daily activities, work, and quality of life. As of 2019, migraine represents the seventh-highest specific cause of disability, and the highest cause of disability-adjusted life years among females aged 15–49, globally [3–5].

Spontaneous abortion (SAB) is defined as pregnancy loss before 20 weeks of gestation, occurring in about 20% of recognized pregnancies [6, 7]. It is a significant physical and psychological stressor and has been associated with subsequent obstetrical complications, anxiety, depression, and post-traumatic stress [8–11].

Pathophysiological mechanisms that could underlie an association between migraine and SAB are poorly understood [12]. Common proposed pathways include central nervous system excitability, autoimmune conditions, and autonomic dysfunction [12–14]. Moreover, migraine may be a marker of chronic systemic inflammation and subsequent endothelial damage, both associated with recurrent SAB [12, 15].

Migraine is associated with adverse pregnancy outcomes such as hypertensive disorders, low birth weight and preterm birth [13], but studies of the association between migraine and SAB risk have been inconsistent, potentially due to methodological differences. In a 2005 case-control study, severe migraine during pregnancy was associated with lower odds of threatened abortion compared with no migraine (odds ratio 0.7, 95% confidence interval [CI]: 0.6–0.9) [16]. Conversely, recent cohort studies have shown a modest increase in SAB risk among women with a history of migraine, independent of migraine treatment. A Danish registry study found that a history of migraine (vs. none) was associated with a 10% increased risk of SAB (95% CI: 1.05–1.15) [12]. However, the study relied on hospital or outpatient clinic visits to ascertain SAB, and consequently missed most SABs that occur before 8 weeks of gestation, which constitute more than 50% of all recognized SABs [17–19]. Early and late SABs may have differing risk factors [20], and this study may not have been able to detect an association between migraine and early losses.

Few studies have examined the association between migraine medications and SAB. A nested case-control study and a meta-analysis found that triptan use among women with migraine was not appreciably associated with risk of SAB. However, migraineurs who used either triptans or nonsteroidal anti-inflammatory drugs had nearly three times the risk of SAB compared with non-migraineurs, implying that the underlying pathophysiology of migraine itself, rather than the treatment, increases SAB risk [21, 22].

Herein, we examined the association of self-reported diagnoses of preconception migraine and medication use with SAB risk. Pre- and peri-conceptional exposures may be particularly relevant for early SAB, for which known risk factors are limited [23]. We hypothesized that individuals with a history of preconception migraine would be at increased risk of SAB, but that migraine medication use would not be associated with a further increase in SAB risk.

## Methods

### Study population

We used data from Pregnancy Study Online (PRESTO), an ongoing online prospective study of the effects of lifestyle, dietary, and medical factors on fertility that began in June 2013. Study methods have been described in detail elsewhere [24]. Briefly, eligible participants self-identified as female, are 21–45 years of age, residents of the United States or Canada, and not using contraception or receiving fertility treatment. Participants complete an online baseline questionnaire eliciting information on demographics, lifestyle, and medical and reproductive histories. They then complete follow-up questionnaires every 8 weeks for up to 12 months to ascertain pregnancy status. Participants who report a conception are invited to complete additional questionnaires in early pregnancy (~ 8 weeks of gestation) and late pregnancy (~ 32 weeks of gestation).

### Assessment of exposure

We defined those with a history of migraine as participants with a reported diagnosis of migraine or use of a medication to treat migraine before conception (estimated as 14 days after the date of last menstrual period, LMP). We ascertained migraine history and medication use from self-administered online questionnaires at baseline and during follow-up. At baseline, participants reported whether they had ever been diagnosed with “migraine headaches” and, if so, how many migraines they had experienced in the past 4 weeks (none, one, two, more than two). On baseline and bimonthly follow-up questionnaires, participants reported migraine medication use in the 4 weeks prior to each questionnaire (yes/no), and medication use frequency (i.e., when experiencing symptoms, daily, or daily plus occasional extra use due to symptoms). Participants who completed the questionnaire after April 2016 also reported the name of their most recent migraine medication, if any. We also identified individuals taking migraine medications from a write-in field for pain medication. Participants who reported migraine as the indication for their use of pain relievers, or who provided the name of pain-relieving medication in the migraine medication field, were classified as users of pain-relieving medication for migraine.

We ascertained changes in the use of migraine medication on bimonthly follow-up questionnaires. In our analyses, we prioritized data on migraine medication use from the most recent follow-up questionnaire before estimated conception, as this was deemed to be the most etiologically relevant period of exposure. As we sent preconception questionnaires to participants every 8 weeks, the time between exposure assessment (questionnaire completion) and estimated conception date varied among participants. A timeline of exposure assessment relative to conception is presented in Fig. 2. Recent (previous 4 weeks) migraine medication use was assessed 0–8 weeks before conception for 75% of participants, after conception (before pregnancy detection) for 10% of participants, and more than 8 weeks before conception (participant skipped questionnaire(s)) for 15% of participants.

We classified responses by active ingredient (anticonvulsant, antidepressant, acetaminophen, aspirin, beta blocker, calcium channel blocker, calcitonin gene-related peptide (CGRP) antagonist, combination analgesic/caffeine, muscle relaxant, non-aspirin non-steroidal anti-inflammatory drugs (NSAIDs), opioid, triptan, steroid, other, unknown). We classified pain medications by active ingredient (acetaminophen, aspirin, non-aspirin NSAIDs, and opioid). Pain medications were not mutually exclusive. We classified combination analgesic-caffeine medications by their specific pain-relieving ingredient. We additionally adjusted the pain medication analyses for the participant-reported number of pain medication pills used in the previous month.

We further classified participants who reported medication use on their most recent follow-up questionnaire before conception into three mutually exclusive categories, according to type of medication: prescription migraine prophylaxis medication, prescription migraine treatment, and over-the-counter (OTC) migraine medication. Medication type may indicate migraine severity, with use of a daily preventive medication indicating higher migraine severity, followed by episodic prescription treatment and over-the-counter treatment. We categorized participants who reported use of multiple medication types into the highest hypothesized severity group indicated by their responses.

### Assessment of outcome

We defined SAB as pregnancy loss before 20 completed weeks of gestation, captured by questionnaire responses of “miscarriage”, “chemical pregnancy”, or “blighted ovum”. On follow-up questionnaires, participants reported the dates and results of home pregnancy tests, including negative and positive tests. Pregnant participants were asked how they detected their pregnancy (home pregnancy test, urine test at doctor’s appointment, etc.). Participants reported pregnancy losses, including date of loss, on bimonthly follow-up questionnaires and early and late pregnancy questionnaires. We calculated gestational weeks from the pregnancy LMP date.

If participants were lost to follow-up after reporting a pregnancy, we attempted to determine the pregnancy outcome by contacting them via alternative channels. These included email, phone, searching social media and baby registries, using data shared by participants from fertility tracking apps, and linking participant data to birth registries in states with the highest numbers of PRESTO participants (MA, CA, NY, FL, MI, TX, PA, and OH). If we were able to contact participants, we asked them to provide information on pregnancy status, including date of pregnancy loss, if applicable. If we identified a birth in a birth registry that corresponded to an LMP date during the study period, we assumed there was no pregnancy loss. If we were unable to contact participants, we censored them at completion of their last questionnaire.

### Assessment of covariates

We ascertained values of covariates from information collected on the baseline questionnaire. Covariates included maternal age, partner age, education, body mass index, physical activity, smoking, alcohol intake, caffeine intake, polycystic ovary syndrome (PCOS), endometriosis, irregular menstrual cycles, history of anxiety or depression, stress (Perceived Stress Scale [PSS]--10 score) [25], and depressive symptoms (Major Depression Inventory [MDI] score) [26].

### Statistical analysis

**Fig. 1:**
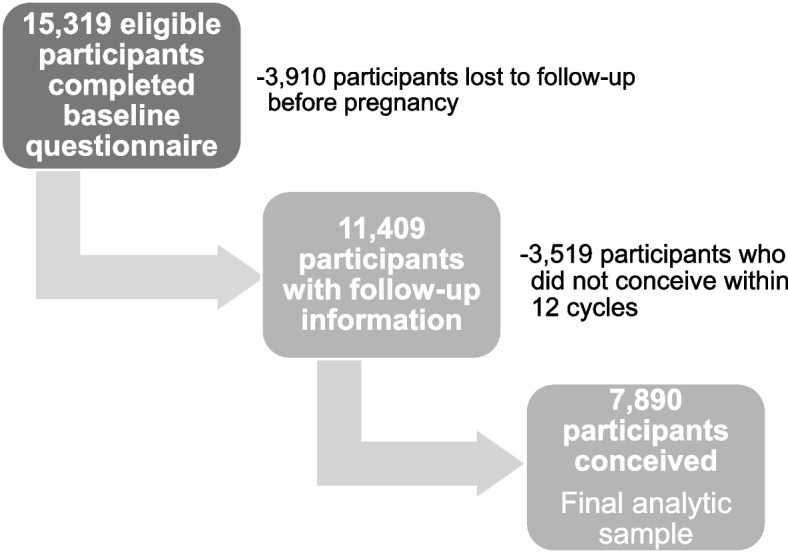
Pregnancy Study Online study population and exclusions, June 2013–September 2021

From June 2013 through September 2021, 15,319 eligible female participants enrolled and completed the baseline questionnaire. We excluded 3910 participants (26%) who completed the baseline questionnaire but were lost to follow-up before identification of pregnancy, and 3519 (23%) who did not conceive within 12 months of follow-up (Fig. 1). Participants who were lost to follow-up tended to be younger, had lower educational attainment and household income, and reported higher levels of stress than individuals who were not lost to follow-up. Loss to follow-up was similar among participants with and without a diagnosis of migraine at baseline (26% of each group). The total analytic sample was 7890 participants (Fig. 1).

**Fig. 2:**
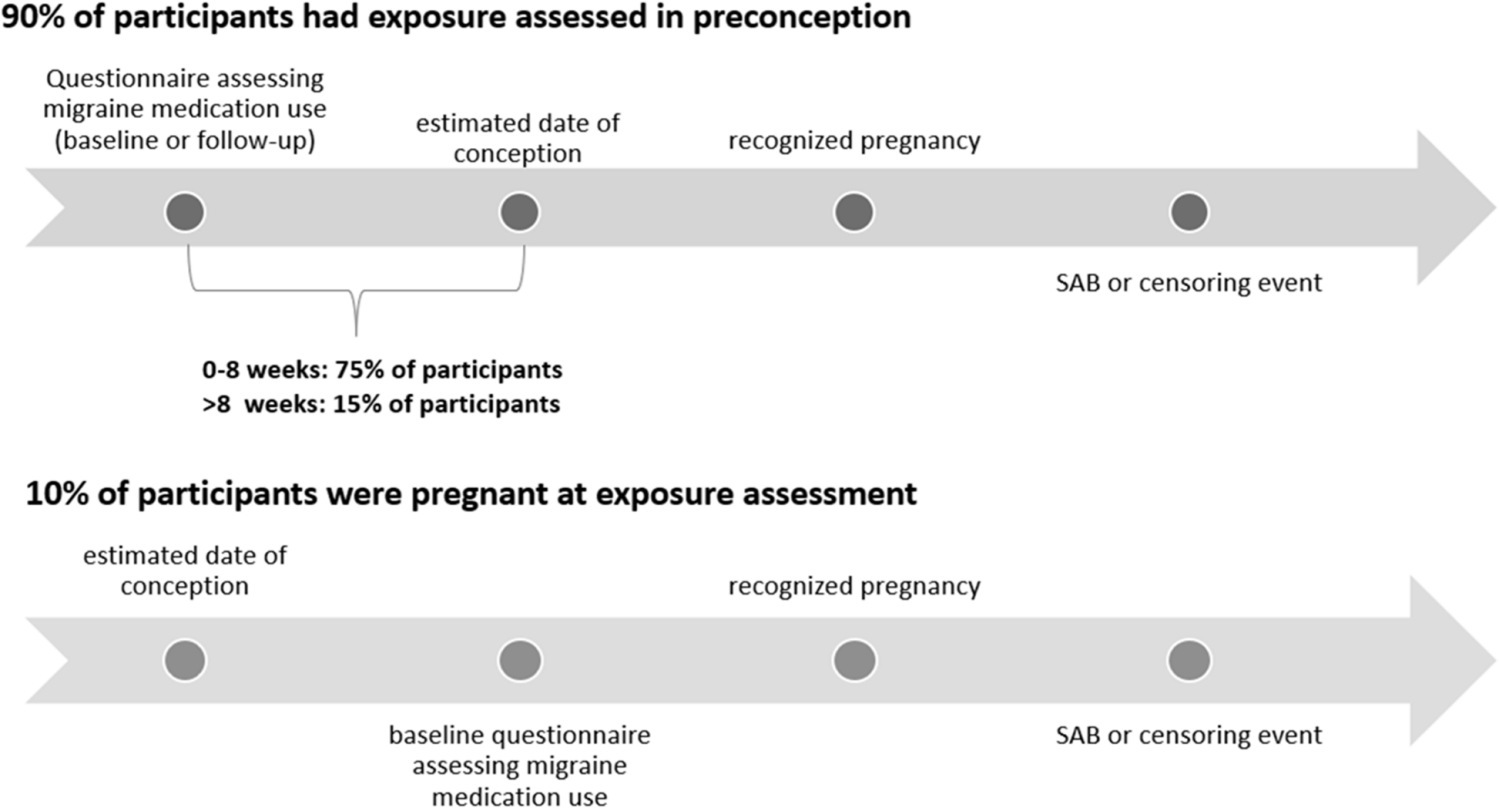
Assessment of migraine medication use relative to exposure.*2*

We used descriptive statistics to examine associations between migraine diagnosis, medication use, and baseline covariates. We used multiple imputation to impute missing data on covariates applying fully conditional specification methods [27]. We created 20 imputed datasets and statistically combined coefficient and standard error estimates across datasets. No participants were missing data on migraine status. As pain medication indication and specific migraine medication were not added to the questionnaire until 2016, this information was missing for many medication users. We limited pain medication analyses to individuals who provided an indication (*n* = 1683). Missingness for all covariates was < 1%, except for physical activity (1.2%) and household income (2.7%). For 1% of participants, we imputed missing gestational weeks at pregnancy loss or censoring events.

We estimated gestational weeks at SAB based on participant-reported weeks of pregnancy duration. When this information was unavailable, we estimated gestational weeks at SAB based on the pregnancy end date and pregnancy due date, using the following formula: pregnancy end date-(pregnancy due date-280)/7. When no pregnancy due date was available, we estimated gestational weeks at SAB using an alternate formula: (pregnancy end date-LMP date)/7. We applied the Andersen-Gill data structure [28] with one observation per week of gestation, starting at gestational week of pregnancy detection (when available) or 4 weeks (i.e., median gestation at first pregnancy detection in the cohort) and ending at SAB, 20 weeks of gestation, or other censoring event (e.g., ectopic pregnancy or induced abortion, study withdrawal, or completion of last questionnaire if lost to follow up), whichever came first.

We used Cox proportional hazards regression with gestational weeks as the time scale to estimate hazard ratios (HRs) and 95% confidence intervals (CIs) for the association between migraine and SAB [29]. We selected confounders based on a literature review and a directed acyclic graph. In the final models we adjusted for the following baseline confounders: age (< 25, 25–29, 30–34, ≥35 years), partner age (continuous), education (< 16 vs. ≥16 years), body mass index (BMI, kg/m^2^, continuous), current smoker at baseline (yes/no), alcohol intake (0, 1–6, 7–13, ≥14 drinks per week), caffeine intake (mg/day, continuous), recent irregular menstrual cycles (yes/no), physical activity (total metabolic equivalents of activity per week [30, 31], continuous), stress (perceived stress scale (PSS-10) score [25], continuous), depressive symptoms (Major Depression Inventory (MDI) [26] score, continuous), and diagnoses of PCOS, endometriosis, anxiety, or depression (yes/no).

We examined the association between any reported migraine (ever vs. never) and migraine frequency (0, 1, 2, > 2) in the 4 weeks before baseline and SAB. Based on the most recent follow-up questionnaire before the estimated date of conception, we examined the following exposures: use of any migraine medication in the last 4 weeks (yes, no), use of specific medication types (prescription preventive medication, prescription treatment medication, over-the-counter medication), and use of migraine medications containing specific ingredients (analgesic(s) and caffeine, triptans). We compared these data to responses from individuals who did not report migraine at baseline or during follow-up. We also subcategorized early and late SAB (< 8 vs. ≥8 weeks), to determine whether any potential association between migraine and SAB was stronger for early SAB. We hypothesized that preconception exposures would contribute to an increase in early, rather than late, SAB [23, 32].

To assess confounding by indication, we compared SAB risk among users of pain medications for migraine or other indications with non-users of pain medications in the previous 4 weeks. We adjusted for potential confounding variables listed above, as well as for the total number of pain medication pills the participant reported using in the previous 4 weeks (for any indication, as a proxy for medication dose). We also adjusted results for use of medication to treat urinary tract infections, pelvic inflammatory disease, hypertension, thyroid disorders, diabetes, fibroids, PCOS, depression, anxiety, hay fever, endometriosis, acid reflux, or use of antibiotics reported on the same questionnaire (yes to any, no).

## Results

### Overall results

Of the 7890 pregnant participants in the study population, 1537 (19%) experienced SAB during 95,937 gestational weeks of observation. Median gestational weeks at SAB was 6 (range 3–19 weeks). Overall, 21% of participants (*n* = 1683) reported having a preconception diagnosis of migraine or migraine medication use. Of the 1633 participants who reported a history of migraine at baseline (50 participants reported a new diagnosis of migraine during preconception follow-up), 42% reported no migraine in four weeks before baseline, 28% reported one migraine, 14% reported two migraines, and 16% reported more than two migraines.

Table 1 shows the distribution of covariates at baseline by preconception migraine status and preconception migraine medication status. There was little difference in demographic characteristics between migraineurs and non-migraineurs. Migraineurs who took medication for migraine had slightly lower educational attainment and household income than non-migraineurs. Those taking migraine medication also were more likely to have a history of SAB than participants without migraine;32% of migraineurs who took medication, 26% of migraineurs who did not take medication, and 25% of non-migraineurs reported a history of SAB. Participants with migraine were more likely than those without migraine to report a history of infertility and diagnoses of PCOS, endometriosis, or anxiety disorders, regardless of medication use. Although many lifestyle factors measured at baseline are common migraine triggers (alcohol use, stress, physical activity, caffeine), migraineurs did not have an appreciably different prevalence of these triggers compared to non-migraineurs.

**Table 1:** Baseline characteristics by migraine status^a^ of 7,890 PRESTO participants who conceived, 2013–2021*.*

**Characteristic**	**No migraine (*N*** **= 6207, 79%)**	**Migraine (N = 1683, 21%)**	
		**No medication for migraine (*N*** **= 1021, 61%)**	**Medication for migraine (*N*** **= 662, 39%)**
**Demographic**			
Age (%)			
*25 years*	7	6	8
*25–29 years*	40	38	39
*30–34 years*	41	43	40
*≥35 years*	12	13	13
Partner age (mean)	32	32	32
Less than college degree (%)	19	22	26
Income <$50,000 (%)	14	14	18
Region of residence (%)			
*Northeast*	24	24	22
*South*	22	22	24
*Midwest*	22	22	25
*West*	16	16	17
*Canada*	16	16	12
**Medical History**			
Irregular menstrual cycles (%)	13	15	15
Parous (%)	33	32	37
History of spontaneous abortion (%)	25	26	32
History of infertility^b^ (%)	7	9	11
History of diagnosed polycystic ovary syndrome	5	8	10
History of diagnosed endometriosis (%)	2	5	6
History of diagnosed anxiety disorder (%)	23	34	37
History of diagnosed depression (%)	22	32	33
Body mass index (kg/m^2^, mean)	27	27	28
**Lifestyle**			
Multivitamin use (%)	86	84	84
Smoking (%)			
*Ever smoker*	18	22	33
*Current smoker*	4	4	6
Alcohol intake, drinks/week (%)			
*< 1*	26	27	31
*1–6*	59	62	58
*7–14*	13	8	8
*>14*	3	3	2
Physical activity (total metabolic equivalent hours per week, mean)	35	34	33
Stress (Perceived Stress Scale-10 score, mean)	16	16	17
Depressive symptoms (Major Depression Inventory Score, mean)	10	11	13
Caffeine intake (mg/day, mean)	126	135	140

^a^Migraine defined as any participant report of a history of migraine at baseline or migraine medication use at baseline or during follow-up.^b^History of infertility defined as a participant report of a previous pregnancy attempt over > 12 cycles

A preconception history of migraine was not appreciably associated with SAB risk (HR = 1.03, 95% CI: 0.91–1.16) (Table 2). Results were similar for early and late SABs. Migraine frequency also was not appreciably associated with SAB risk: HRs for 0, 1, 2, and > 2 migraine episodes in the 4 weeks before baseline (compared with no history of migraine) were 1.03 (95% CI: 0.86–1.23), 0.93 (95% CI: 0.74–1.16), 1.10 (95% CI: 0.82–1.37), and 1.14 (95% CI: 0.86–1.49), respectively.

**Table 2:** Association between migraine and spontaneous abortion (*n* = 7890)

	SABs	Pregnancies	Crude HR	Adjusted HR^**a**^
**Preconception Migraines**				
No	1199	6207	Ref	Ref
Yes	338	1683	1.05 (0.93–1.18)	1.03 (0.91–1.16)
**Earlier losses (< 8 weeks)**				
No	808	5684	Ref	Ref
Yes	228	1544	1.04 (0.90–1.20)	1.01 (0.87–1.18)
**Later losses (≥8 weeks)**				
No	391	4856	Ref	Ref
Yes	110	1300	1.06 (0.86–1.32)	1.06 (0.85–1.32)
**Migraine frequency**^**b**^				
0	141	693	1.05 (0.88–1.25)	1.03 (0.86–1.23)
1	84	463	0.94 (0.75–1.17)	0.93 (0.74–1.16)
2	48	222	1.13 (0.85–1.51)	1.10 (0.82–1.37)
> 2	55	255	1.14 (0.87–1.50)	1.14 (0.86–1.49)

^a^Adjusted for baseline age, partner age, education, BMI, smoking, alcohol use, polycystic ovarian syndrome, endometriosis, anxiety, depression, MDI score, physical activity, stress, caffeine before intake. ^b^Assessed during the 4 weeks prior to baseline only

### Migraine medication use

Approximately half (*n* = 807, 48%) of the 1683 migraineurs reported medication use at baseline or during preconception follow-up. Migraineurs who reported no preconception migraine medication use did not have a higher risk of SAB than non-migraineurs (HR = 0.96, 95% CI: 0.82–1.13) (Table 3). Migraineurs who used medication in the past 4 weeks on the most recent questionnaire before conception had a slightly higher SAB risk (HR = 1.14, 95% CI: 0.96–1.36). Daily use of migraine medication was associated with an increased risk of SAB (HR = 1.38, 95% CI: 0.81–2.35), whereas less than daily use of migraine medication was not (HR = 1.09, 95% CI: 0.90–1.32).

**Table 3:** Association between migraine medication use and spontaneous abortion (n = 7890)^a^

	# SABs	# Pregnancies	Crude HR	Adjusted HR^**b**^
**Migraine medication use**				
Non-migraineur	1199	6207	Ref	Ref
Migraineur, no medication use	168	893	0.97 (0.83–1.14)	0.96 (0.82–1.13)
Migraineur, medication use	145	662	1.15 (0.97–1.37)	1.14 (0.96–1.36)
**Frequency of use**				
Less than daily	121	572	1.10 (0.91–1.32)	1.08 (0.90–1.32)
Daily	14	55	1.38 (0.81–2.33)	1.38 (0.81–2.35)
**Type**				
Over-the-counter treatment	102	456	1.17 (0.94–1.45)	1.16 (0.93–1.44)
Prescription treatment	32	165	1.04 (0.71–1.53)	1.04 (0.71–1.53)
Prescription prophylaxis	11	41	1.41 (0.70–2.83)	1.43 (0.72–2.84)
I**ngredients**				
Analgesic(s), and caffeine	31	119	1.38 (0.97–1.98)	1.42 (0.99–2.04
Triptans	19	77	1.30 (0.83–2.05)	1.30 (0.83–2.07)

^a^Assessed in the last follow-up questionnaire before conception .^b^Adjusted for baseline age, partner age, education, BMI, smoking, alcohol use, polycystic ovarian syn

While use of prescription migraine prophylaxis medication, indicative of the most severe cases, was associated with increased risk of SAB (HR = 1.43, 95% CI: 0.72–2.84), there were only 11 SABs among exposed participants. The HRs for use of prescription (episodic) or over-the-counter episodic medication were 1.04 (95% CI: 0.71–1.53) and 1.16 (95% CI: 0.93–1.44), respectively. Due to the variety of write-in migraine medications reported, we could analyze only the association between the two most frequently-reported specific active ingredients and SAB. The HR for use of analgesic/caffeine medication was 1.42 (95% CI: 0.99–2.04) and the HR for use of triptans was 1.30 (95% CI: 0.83–2.07).

Table 4 shows HRs for the association between migraine medication use and SAB risk, stratified by the timing of exposure assessment. HRs were similar when the exposure was assessed post-conception (HR = 1.06, 95% CI: 0.61–1.82), 0–4 weeks before conception (HR = 1.13, 95% CI: 0.88–1.43), and 5–8 weeks before conception (HR = 1.14, 95% CI: 0.81–1.61). The association between migraine medication use and SAB was strongest when exposure was assessed >8 weeks before conception (HR = 1.43, 95% CI: 0.87–2.37).

**Table 4:** Association between preconception migraine medication use and spontaneous abortion, by timing of medication use assessment (*n* = 7012)*. *

	SABs	Pregnancies	Crude HR	Adjusted HR^**a**^
**Migraine medication use, assessed after conception**^**b**^				
No (non-migraineur)	125	627	Ref	Ref
Yes	15	72	1.10 (0.64–1.87)	1.06 (0.61–1.82)
**Migraine medication use, assessed 0–4 weeks before conception**				
No (non-migraineur)	631	3102	Ref	Ref
Yes	72	311	1.15 (0.90–1.47)	1.13 (0.88–1.43)
**Migraine medication use, assessed 5–8 weeks before conception**				
No (non-migraineur)	344	1572	Ref	Ref
Yes	38	156	1.11 (0.80–1.56)	1.14 (0.81–1.61)
**Migraine medication use, assessed > 8 weeks before conception**				
No (non-migraineur)	906	1049	Ref	Ref
Yes	20	123	1.53 (0.95–2.47)	1.43 (0.87–2.37)

^a^Adjusted for baseline age, partner age, education, BMI, smoking, alcohol use, polycystic ovarian syndrome, endometriosis, anxiety, depression, MDI score, physical activity, stress, caffeine intake.^b^Participant was pregnant, but unaware

### Pain medication use

Most participants (*n* = 5362, 68%) reported pain medication use on their most recent follow-up questionnaire before conception, and 7% reported migraine as the indication for using pain medication. The HR for use of pain medication for indications other than migraine was 0.99 (95% CI: 0.84–1.17), while the HR for use of pain medication for migraine was 1.15, (95% CI: 0.84–1.47) (Table 5). Generally, use of specific pain medications for migraine or other indications was not meaningfully associated with SAB risk. Use of aspirin for migraine was associated with a slight increased risk of SAB (HR = 1.21, 95% CI: 0.82–1.79), while use of aspirin for other indications was associated with a decreased risk of SAB (HR = 0.76, 95% CI: 0.42–1.38), compared with no pain medication use.

**Table 5:** Association between use of pain medication for migraine and spontaneous abortion (*n* = 7890)^a^

	SABs	Pregnancies	Crude HR	Adjusted HR^**b**^	Adjusted HR^**c**^
**Pain medication overall**					
No pain medication	492	2483	Ref	Ref	Ref
Pain medication for other indications	279	1301	1.11 (0.95–1.28)	0.99 (0.84–1.16)	0.99 (0.84–1.17)
Pain medication for migraine	87	351	1.32 (1.05–1.66)	1.16 (0.91–1.48)	1.15 (0.84–1.47)
**Specific pain medication**					
No pain medication	492	2483	Ref	Ref	Ref
**NSAID (non-aspirin)**					
Use for other indications	189	869	1.08 (0.90–1.28)	0.93 (0.77–1.13)	0.93 (0.76–1.12)
Use for migraine	43	200	1.12 (0.82–1.52)	0.94(0.68–1.31)	0.95 (0.68–1.32)
**Acetaminophen**					
Use for other indications	143	636	1.16 (0.96–1.40)	1.05 (0.86–1.28)	1.06 (0.86–1.30)
Use for migraine	58	234	1.30 (0.99–1.71)	1.15 (0.86–1.54)	1.12 (0.83–1.51)
**Aspirin**					
Use for other indications	13	76	0.86 (0.50–1.49)	0.78 (0.43–1.39)	0.76 (0.42–1.38)
Use for migraine	37	145	1.34 (0.96–1.87)	1.20 (0.81–1.77)	1.21 (0.82–1.79)

^a^Assessment based on the most recent follow-up questionnaire before conception.^b^Adjusted for baseline age, partner age, education, BMI, smoking, alcohol use, polycystic ovarian syndrome, endometriosis, anxiety, depression, MDI score, physical activity, stress, caffeine intake.^c^Further adjusted for number of pain medication pills and use of any other medication on the follow-up questionnaire closest to conception

## Discussion

In this prospective cohort study of pregnancy planners residing in the U.S. and Canada, a preconception history of migraine was not appreciably associated with SAB risk. Use of migraine medication, however, was associated with a modest increase in SAB risk overall. The highest risks occurred among those taking migraine medications daily, receiving prescription migraine prophylaxis, or taking combination analgesic and caffeine medications. Our findings suggest that while migraine overall may not be associated with SAB, patterns of medication use suggesting more severe migraine may be associated with greater SAB risk.

Reported use of prescription migraine prophylaxis medication at the most recent follow-up questionnaire before conception, which may indicate increased migraine frequency or severity, was associated with the greatest increase in SAB risk of any subgroup analyzed. This may be attributable to the medications used for migraine prophylaxis, which differ from medications used to treat acute migraine attacks. However, there were too few users of migraine prophylaxis medications in our study population to separately analyze the impact of medications by type. Daily migraine medication use at baseline also was associated with increased SAB risk.

We observed a slightly elevated risk of SAB among users of many active ingredients, including aspirin to treat migraine, triptans, and combination analgesic/caffeine medication. Thus, migraine requiring medication were associated with an increase in SAB risk, independent of ingredient. Many commonly used migraine medications contained a combination of analgesics (often acetaminophen and aspirin), contributing to substantial overlap in individuals across these analyses.

Notably, we found no meaningful association between use of non-aspirin NSAID use and SAB. The literature on NSAID use and SAB overall is inconclusive. While a German case series found no suggestion of a teratological effect of naproxen in the first trimester, a meta-analysis of NSAIDs in pregnancy found that use around the time of conception was associated with an increased risk of miscarriage, although only two studies were included [33, 34].

Previous studies have reported an increase in SAB risk among individuals taking medication specifically for migraine. In one study, migraineurs who used ibuprofen to treat their migraine had triple the risk of SAB, compared to non-migraineurs [21]. *Marchenko* et al. found an increased risk of SAB among triptan users compared with non-migraineurs.^22^In our study, triptan use was associated with modestly increased risk of SAB (HR = 1.30, 95% CI: 0.83–2.07), although to a lesser extent than reported by Marchenko et al.[22]Valproate is indicated for migraine prophylaxis and known to have fetotoxic effects [35], however use of valproate was extremely rare in our study. While caffeine consumption during pregnancy in moderate to high amounts (> 200 mg per day) has also been associated with an increased risk of SAB [36], most migraine medications with caffeine contain 65–130 mg per dose, which is unlikely to meaningfully increase SAB risk if taken intermittently. Migraine-specific medications such as combination analgesic/caffeine and triptans may be used for migraine that does not respond to non-aspirin NSAIDs. This could result in confounding by migraine severity, which we did not measure directly.

While migraine medication use was relatively common in our study population, many migraine medication users did not provide the name of their specific medication. Therefore, we were unable to analyze several additional types of medications used for migraine, such as beta blockers and antidepressants. Such missing data could lead to bias if missingness were related to medication type and SAB. However, this cause of bias is unlikely in our study, as information was collected prospectively and most missingness was effectively random (i.e., related to calendar year of questionnaire completion, as specific migraine medication questions were added more than 3 years after study commencement). Missing data reduced our sample size for several medication use analyses, resulting in limited precision and greater uncertainty in these findings.

Another concern is our reliance on self-reported migraine status. Individuals with more frequent or more severe migraine may be more likely to receive a diagnosis, while individuals whose migraine is less frequent or well-controlled with OTC medications may not receive a diagnosis, despite their underlying migraine pathophysiology. We did not have sufficient information to use International Classification of Headache Disorders (ICHD) diagnostic criteria [37]. While we did not perform a validation study in this population, a previous study comparing self-reported physician diagnosed migraine and ICHD-2 criteria found high agreement between these measures in a pregnancy cohort [ 38]. Ultimately, while under-ascertainment of migraine status was probable, our exposure measurement likely accurately categorized individuals with severe and recent migraine.

We were unable to distinguish migraine triggered by hormonal fluctuations, known as menstrual migraine, from migraine triggered by other factors, such as diet. Menstrual migraine may be more likely to be related to SAB, due to the possible mechanistic role of ovarian hormones in their pathophysiology [39]. We also did not collect information on whether participants experienced migraine with or without aura. Migraine with aura may more likely relate to vascular sensitivity [40, 41], and therefore SAB, given the role of vascular and inflammatory factors in implantation [42].

We ascertained migraine medication from the most recent follow-up questionnaire completed before conception to isolate the most etiologically relevant time window of exposure (peri-conception). Many teratogenic exposures occur early in pregnancy, often before pregnancy detection, and most SABs in the cohort occurred within the first 8 weeks of pregnancy [23]. Additionally, many individuals seek to decrease their migraine medication use after pregnancy detection, so medication use reported after pregnancy detection may not reflect actual exposure during the first few weeks of pregnancy [43]. In general, misspecification of the etiologically relevant time window for exposure would be expected to attenuate our associations [44]. If the underlying mechanism is shared pathophysiology for migraine (or migraine severity requiring medication use) and SAB, this mechanism would be less time-specific, minimizing bias. While some degree of outcome misclassification is inevitable, given the unobservable nature of the exact timing of conception, 95% of PRESTO participants reported at-home pregnancy testing. The median timing of pregnancy detection in the cohort was 4 weeks, indicating that our study likely captured earlier losses than studies that rely on hospital visits or medical records.

Although we collected extensive information on participant demographic, lifestyle, and medical characteristics, unmeasured or residual confounding is possible. While we adjusted for several conditions known to be comorbid with migraine, residual confounding is possible given the frequency with which migraine co-occurs with other medical conditions and their associated treatments [43]. Unmeasured or residual confounding by factors related to access to or willingness to seek health care is also possible.

Bias due to self-selection in this internet-based prospective cohort study is unlikely. Participants are not pregnant at enrollment; therefore, we would not expect differential enrollment by exposure and future pregnancy status. Previous research has shown that internet-based recruitment methods are not more prone to selection bias than traditional methods [45].

## Conclusion

Overall, this prospective cohort study provides an estimate of the associations of preconception migraine history and medication use with SAB risk, using self-reported exposure, confounder, and outcome information not available in registry studies. Migraineurs who have never received a formal migraine diagnosis and exclusively use OTC medications may not be captured through medical record or registry data sources. The present prospective study of pregnancy planners addresses this limitation.

Our findings indicate that a history of migraine among females is not associated with SAB risk to a meaningful extent, but routine use of medication for migraine during the preconception period may be associated with greater SAB risk. This increased risk is likely due to more severe underlying vascular pathophysiology as opposed to risk from medications.

## Data Availability

The datasets generated and/or analyzed during the current study are not publicly available due to participant confidentiality.

## Abbreviations

BMI: body mass index
CGRP: calcitonin gene-related peptide
CI: confidence interval
HR: hazard ratio
ICHD: International Classification of Headache Disorders
LMP: last menstrual period
MDI: Major Depression Inventory
NSAID: non-steroidal anti-inflammatory
OTC: over-the-counter
PCOS: polycystic ovarysyndrome
PRESTO: Pregnancy Study Online
PSS: Perceived Stress Scale
SAB: spontaneous abortion
US: United States
USD: United States dollar
